# Molecular-biomechanical phenotyping of ankle osteoarthritis: from synovial biomarkers to weight-bearing digital signatures

**DOI:** 10.3389/fmed.2026.1931266

**Published:** 2026-08-13

**Authors:** Xueqing Han, Yufei Long, Weiwei He

**Affiliations:** Department of Hand and Foot Surgery, Neijiang Hospital of Traditional Chinese Medicine, Neijiang, China

**Keywords:** ankle osteoarthritis, center of pressure, distance map, gait analysis, plantar pressure, post-traumatic osteoarthritis, precision medicine, synovial fluid biomarkers

## Abstract

Ankle osteoarthritis (OA) is commonly staged by pain, deformity, and radiographic joint-space loss, but this morphology-first view leaves much of its clinical heterogeneity unexplained. An ankle-specific precision framework is biologically and technically plausible because ankle OA is strongly enriched for post-traumatic mechanisms, has cartilage and synovial biology that differs from knee OA, and can be measured with weight-bearing structural and digital functional tools. Comparative cartilage studies show joint-dependent differences in catabolic susceptibility, matrix synthesis, transport, cell-matrix organization, and injury response. Human synovial-fluid and synovial-transcriptomic studies identify local inflammatory, remodeling, and WNT-related signals, while weight-bearing computed tomography (WBCT), automated three-dimensional alignment metrics, distance maps, gait analysis, plantar pressure, center of pressure (COP), and ankle-specific activity monitoring quantify structural and functional load signatures. This Mini Review integrates these data into four provisional, non-mutually-exclusive baseline research phenotypes: inflammatory-dominant, malalignment-dominant, post-traumatic or instability-dominant, and end-stage structural collapse. Post-surgical recovery is treated separately as a longitudinal response trajectory rather than a fifth baseline phenotype. The evidence is comparatively mature for ankle-knee cartilage differences, promising but preliminary for synovial, three-dimensional structural, and laboratory functional signatures, and exploratory for center-of-pressure recovery and wearable trajectories. A validation-ready pathway is proposed around a defined minimum same-patient dataset, serial measurement, a 24-month ankle-specific patient-reported outcome, explicit minimal-important-difference rules, trajectory modeling, calibrated prediction, and external evaluation. The framework is intended to organize prospective cohorts and treatment-response questions; it is not a validated clinical classification or automated treatment selector.

## Introduction

Ankle osteoarthritis (OA) is less prevalent than knee OA, but its individual burden is substantial. A large tertiary-center series showed that ankle arthritis is predominantly post-traumatic, and health-related quality-of-life comparisons suggest that end-stage ankle arthrosis can be as disabling as end-stage hip arthrosis (1, 2). Etiology-focused ankle OA studies and recent reviews reinforce the same message: ankle OA is not simply a rare small-joint variant of generalized OA, but a joint-specific disorder shaped by injury, alignment, instability, and local tissue response (3–6).

The post-traumatic context makes morphology-only staging especially incomplete. Post-traumatic OA is a recognized epidemiologic category across joints, lateral ankle sprain and chronic ankle instability have long-term consequences, and ankle fracture follow-up studies show that residual step-off, fracture-dislocation, and unstable ankle-fracture patterns can be followed by persistent radiographic degeneration (7–12). These data justify a review strategy that links injury timing to later molecular, structural, and functional measurements.

This premise is biologically ankle-specific. Reviews and mechanistic studies comparing ankle and knee joints show that ankle cartilage has distinct homeostatic, matrix, and injury-response properties, including differences in fibronectin-fragment susceptibility, interleukin-1beta responsiveness, proteoglycan synthesis, matrix transport, cell-matrix organization, cartilage degeneration patterns, chondron architecture, and impact response (13–23). Knee-derived biomarker assumptions should therefore be treated as hypotheses rather than defaults.

A parallel measurement shift is occurring in molecular biology, imaging, and biomechanics. Synovitis and WNT biology are central themes in OA pathogenesis more broadly, while ankle-specific studies now show acute post-fracture synovitis, candidate synovial-fluid biomarkers, and synovial transcriptomic dysregulation (24–26). At the same time, weight-bearing computed tomography (WBCT), automated three-dimensional measurement, distance maps, gait analysis, plantar pressure, and center of pressure (COP) trajectories can quantify structural and functional load signatures (27–34).

**Figure 1:**
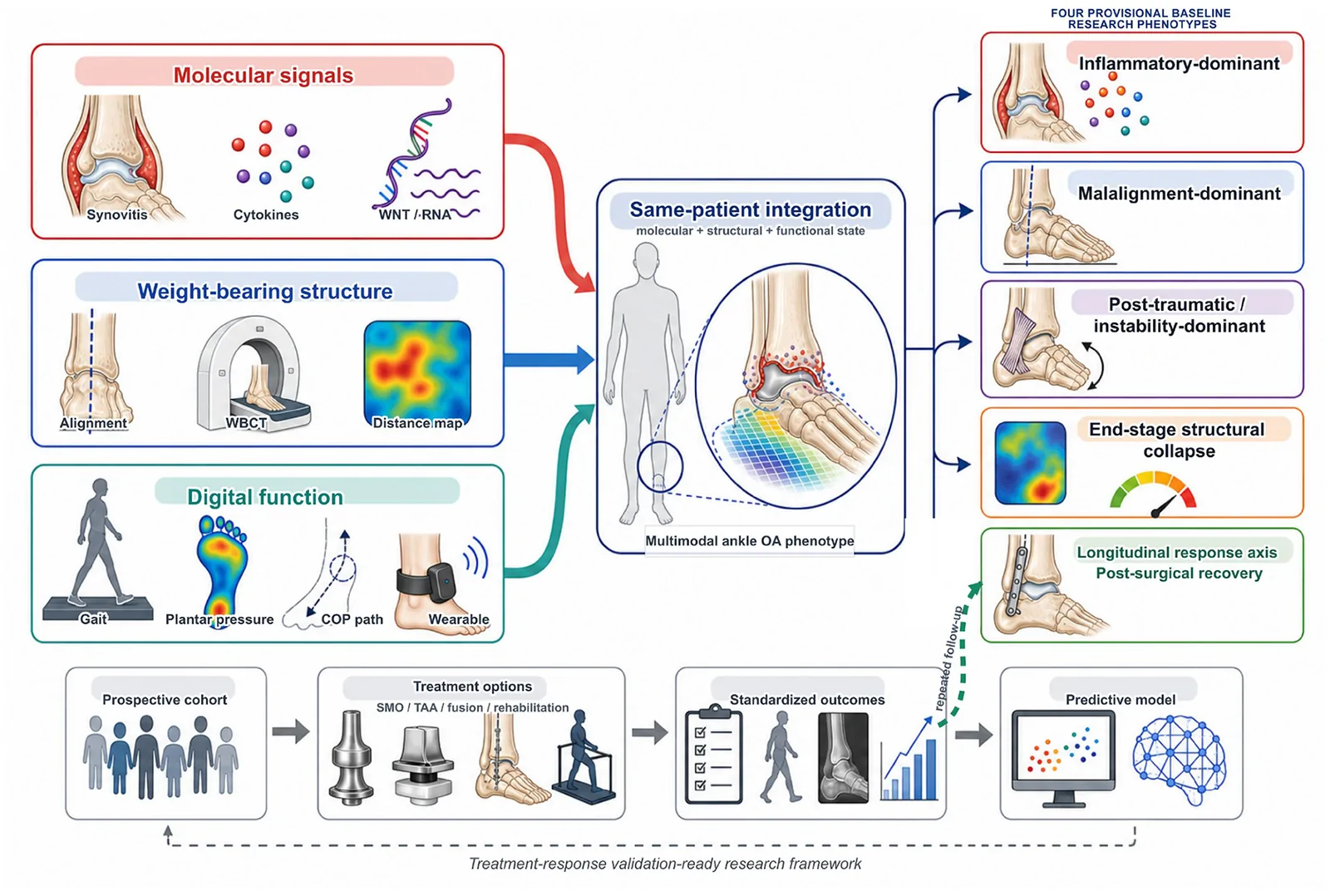
A molecular-biomechanical phenotyping framework for ankle osteoarthritis. Ankle OA is represented as a proposed multimodal disease state shaped by three linked data streams. Synovial and molecular inputs include post-injury synovitis, candidate synovial-fluid biomarkers, and synovial transcriptomic dysregulation. Weight-bearing structural inputs include malalignment, talar rotation, peritalar compensation, automated WBCT metrics, and intra-articular distance-map patterns. Digital functional inputs include laboratory gait metrics, plantar pressure redistribution, COP progression, and wearable-derived activity trajectories. These streams are proposed to define four provisional, non-mutually-exclusive baseline research phenotypes: inflammatory-dominant, malalignment-dominant, post-traumatic or instability-dominant, and end-stage structural collapse. Post-surgical recovery is a separate longitudinal response axis measured from the preoperative state through repeated postoperative visits, not a fifth baseline phenotype. The lower pathway depicts prospective recruitment, treatment, standardized outcomes, and model development with feedback for replication. It is a validation pathway rather than a current treatment-selection algorithm.

This review has three aims. First, it synthesizes ankle-specific biological evidence supporting a joint-specific rather than knee-borrowed model. Second, it links synovial, WBCT, distance-map, gait, plantar pressure, COP, and wearable monitoring studies into one molecular-biomechanical evidence chain. Third, it proposes a phenotype-to-decision research framework. The conceptual architecture is summarized in Figure 1, the key evidence base is organized in Table 1, and the proposed phenotype and longitudinal response framework is summarized in Table 2.

**Table 1:** Key evidence for molecular-biomechanical phenotyping in ankle osteoarthritis.

Domain	References	Design and sample	Key finding	Phenotyping contribution	Evidence maturity and main limitation
Ankle vs knee biology	Kang et al. (15); Huch et al. (16); Eger et al. (17)	Human paired or matched knee–ankle cartilage explants; fibronectin-fragment and IL-1beta challenge; *in vitro* synthesis	Ankle cartilage resisted fibronectin-fragment damage, showed higher cellularity and matrix synthesis, and was less IL-1beta sensitive	Establishes a distinct ankle catabolic and homeostatic baseline	Established *ex vivo*; no clinical outcome
	Fetter et al. (18); Quinn et al. (19)	Human ankle vs. knee cartilage; composition and transport assays; confocal microscopy and stereology	Ankle matrix differs in partitioning, diffusion, and depth-associated chondron organization	Links matrix structure to mediator exposure and mechanical role	Established *ex vivo*; descriptive; no OA cases
	Ayala et al. (23)	Bovine talar dome vs. femoral cartilage; impact plus repetitive articulation	Knee and ankle chondrocytes differ in sensitivity to shear strain	Extends joint–pecific injury biology	Established animal (bovine) tissue, not human; no clinical validation
Molecular	Furman et al. (25)	Acute intra-articular ankle fracture, *n*=6; synovium, synovial fluid, and serum	Fracture increased synovitis, CD68+ macrophage infiltration, and inflammatory cytokines	Defines an early PTOA sampling window	Promising but preliminary very small sample (*n* = 6); acute stage only; no follow-up
	Schmal et al. (26); Schmal et al. (35)	Ankle arthroscopy cohort, *n* = 49; lavage ELISA with clinical and imaging correlation	Aggrecan, BMP-7, bFGF, and CD105 related to symptoms, radiographic stage, and age	Adds stage- and age-aware remodeling signals	Promising but preliminary cross–ectional; single center; one cohort reported twice
	Liu et al. (36)	Post-traumatic ankle OA, *n* = 66; synovial-fluid biomarker study	Lower synovial alpha-MSH tracked disease severity markers	Supports local-fluid severity phenotyping	Promising but preliminary cross–ectional; single candidate marker
	Zou et al. (37)	Ankle PTOA 97 vs. controls 95; serum and synovial-fluid ELISA	Synovial ghrelin tracked severity better than serum	Prioritizes synovial fluid over serum	Promising but preliminary cross–ectional; no treatment-response data
	Matthias et al. (38)	Ankle OA arthroplasty 26 vs. controls 8; prospective bulk RNA–eq	OA synovium showed a distinct transcriptome enriched for ECM remodeling and WNT signaling	Anchors human ankle OA transcriptomics	Promising but preliminary surgically obtained tissue only (selection and stage bias)
	Matthias et al. (39)	Fracture, PTOA, NTOA, and control synovium; bulk RNA–eq with clustering	Fracture and PTOA shared a *WNT7B*- and *IL11*-enriched fingerprint	Supports an active post-traumatic molecular signature rather than injury history alone	Promising but preliminary surgical tissue; clusters not externally replicated
WBCT/3D	Kim et al. (28)	Varus OA 96 vs. control 52 ankles; retrospective WBCT and radiograph study	Internal talar rotation was common in varus OA	Adds axial rotation to the varus phenotype	Promising but preliminary retrospective; varus only; no outcome data
	Krähenbühl et al. (29)	Ankle OA 88 vs. volunteers 27; comparative WBCT subtalar study	Varus OA showed valgus subtalar compensation	Places OA within a peritalar chain	Promising but preliminary cross–ectional; no outcome linkage
	Kvarda et al. (30)	End–tage PTOA 72 vs. controls 20; 2D radiograph vs. 3D WBCT	3D WBCT was more reliable and peritalar compensation was common	Supports 3D structural phenotyping	Promising but preliminary reliability focus; end–tage disease only
	Richter et al. (31); Richter et al. (48)	500 bilateral WBCT cases; automatic vs. manual 3D angular measurement	Early software differed from manual measurement; later validation showed agreement with time savings	Frames automation as feasible but validation-dependent	Promising but preliminary single center and vendor; no outcome linkage
	Tazegul et al. (49)	Healthy 1, mild OA 1, severe OA 1; low-dose WBCT proof of concept	Computational joint–pace quantification was feasible	Introduces digital joint–pace assessment	Exploratory three-case proof of concept (*n* = 3)
	Eatough et al. (50)	Advanced varus OA 44 vs. controls 26; static WBCT joint-angle comparison	Peritalar posture differed by compensation status	Links WBCT posture to compensation	Promising but preliminary static and cross–ectional; advanced varus only
	Efrima et al. (51)	Symptomatic ankle OA 40; distance-map reliability and correlation study	Distance-map sums were reliable and alignment-linked	Defines a digital joint-contact signature	Promising but preliminary reliability and correlation only; no outcome linkage
Digital function	Valderrabano et al. (32)	Unilateral post-traumatic OA 15 vs. controls 15; 3D gait before and after TAA	OA impaired gait; TAA partly restored function at 12 months	Establishes gait as a functional phenotype	Promising but preliminary *n* = 15; single center; 12-month follow-up
	Horisberger et al. (33)	End–tage post-traumatic ankle OA 120; dynamic pedobarography	Painful OA feet redistributed plantar load	Defines load-avoidance pressure patterns	Promising but preliminary cross–ectional; end–tage disease only
	Rouhani et al. (34)	47 subjects across treatment and control groups; in–hoe pressure during 50-m walking	Pressure metrics captured treatment-related differences	Supports pressure-based outcome monitoring	Promising but preliminary small groups; no phenotype stratification
	Zeininger et al. (54)	Symptomatic unilateral OA, *n* = 93; longitudinal force-plate COP analysis before and after TAA	TAA improved COP progression beyond 2 years	Positions COP as a recovery trajectory	Exploratory single center; TAA only; not paired with molecular or structural data
	Shofer et al. (59)	Surgically treated ankle arthritis; step-activity monitoring before surgery and at 6, 12, 24, and 36 months	Objective step activity improved longitudinally and was associated with patient-reported outcomes	Establishes an ankle–pecific wearable recovery trajectory	Exploratory single center; not paired with molecular or structural data
	Kimura et al. (60)	Ankle OA vs. controls; accelerometer-based daily step counts	Ankle OA showed substantially lower daily step counts and lower attainment of activity targets	Supports wearable discrimination, not thresholds	Exploratory cross–ectional; no thresholds or recovery trajectories

Evidence maturity denotes the interpretive weight a study can currently bear for phenotyping, not a formal grade. Established: converging evidence from multiple independent studies. Promising but preliminary: biologically or technically demonstrated, but not reproduced, thresholded, or linked to outcome. Exploratory: feasibility or single-cohort signal only. Formal level-of-evidence and GRADE scores were not assigned, because the included mechanistic, *ex vivo*, animal, reliability, imaging, and longitudinal designs are not commensurable on a single scale, and the level-of-evidence framework is defined for therapeutic, prognostic, and diagnostic clinical questions rather than for *ex vivo* cartilage mechanics. Studies reporting the same cohort (Schmal 2014/2015; Richter 2021/2024) are presented as single entries. BMP, bone morphogenetic protein; COP, center of pressure; ECM, extracellular matrix; *IL11*, interleukin 11; NTOA, non-traumatic osteoarthritis; OA, osteoarthritis; PTOA, post-traumatic osteoarthritis; TAA, total ankle arthroplasty; *WNT7B*, Wnt family member 7B; WBCT, weight-bearing computed tomography.

**Table 2:** Proposed baseline phenotype and longitudinal response framework.

Baseline phenotype or longitudinal response	Molecular or clinical signal	Weight-bearing structural signature	Digital functional signature	Typical scenario	Operational use now	Decision question structured by phenotype or trajectory	Validation priority
Inflammatory–dominant	Synovitis, macrophages, cytokines, candidate markers, WNT activity.	Early injury, subtle malalignment, or limited collapse.	Offloading, early asymmetry, reduced activity tolerance.	Acute intra-articular fracture or early symptomatic PTOA.	Time biofluid sampling with imaging and gait visits.	Who should be followed as molecularly active PTOA?	Link early signals to PTOA onset and treatment response.
Malalignment–dominant	Load-related local markers may accompany deformity.	Varus/valgus alignment, talar rotation, compensation, distance-map asymmetry.	Asymmetric plantar load, altered COP path, compensatory gait.	Varus or valgus ankle OA with peritalar compensation.	Standardize WBCT/distance-map reporting before planning studies.	Is the dominant problem a correctable load path?	Define thresholds predicting SMO, TAA, fusion, or rehabilitation outcomes.
Post–traumatic/instability–dominant	Persisting fracture/PTOA *WNT7B* and *IL11* synovial programme; demonstrable instability.	Rotational malalignment, incongruity, instability-related deformity.	Unstable/asymmetric gait, altered load progression, avoidance.	Active post-traumatic signature (persisting synovial WNT/*IL11* programme and/or mechanical instability), as distinct from burnt–out post–traumatic disease.	Analyze PTOA separately and align injury timing with follow-up.	How should stabilization, correction, and rehabilitation be sequenced?	Track injury-to-PTOA molecular, WBCT, and functional trajectories.
End–tage structural collapse	Chronic inflammatory or catabolic activity as context.	Severe joint–pace loss, distance-map collapse, fixed deformity.	Reduced gait power, plantar offloading, shortened COP path.	Painful advanced OA considered for TAA or fusion.	Integrate residual alignment, compensation, and load capacity.	Which patterns modify expectations after TAA or fusion?	Test multidomain models against implant, fusion, function, and PROMs.
Post–urgical recovery trajectory (longitudinal)	Postoperative remodeling, complications, and rehabilitation exposure.	Corrected/residual alignment; indicated distance maps.	Serial COP, plantar pressure, AOS, and activity at 0, 3, 6, 12, and 24 months.	Follow-up after reconstruction, TAA, fusion, or rehabilitation.	Early sustained, delayed, persistent, or secondary deterioration.	Who needs intensified rehabilitation or reassessment?	Test COP at least 80% of the contralateral value at 12 months and asymmetry below 10% at 24 months; calibrate by procedure and MID.

AOS, Ankle Osteoarthritis Scale; COP, centre of pressure; *IL11*, interleukin 11; MID, minimal important difference; OA, osteoarthritis; PROMs, patient-reported outcome measures; PTOA, post-traumatic osteoarthritis; SMO, supramalleolar osteotomy; TAA, total ankle arthroplasty; WBCT, weight-bearing computed tomography; WNT, Wingless-related integration site (Wnt signalling pathway); *WNT7B*, Wnt family member 7B.

## Why ankle osteoarthritis cannot simply borrow knee osteoarthritis paradigms

The biological foundation for ankle-specific phenotyping begins with the long-recognized difference between ankle and knee OA susceptibility. Early comparative reviews described ankle OA as a distinctive condition, and later molecular work proposed that differences between human joints could arise from joint-specific tissue organization and cellular behavior (13, 14). This background makes it biologically plausible that ankle OA would not share all phenotypes, biomarkers, or treatment-response patterns with knee OA.

Human cartilage studies provide direct support for this distinction. Kang and colleagues showed that ankle cartilage was more resistant than knee cartilage to fibronectin-fragment mediated proteoglycan loss, including in paired donor samples. Huch and colleagues reported higher cellularity and matrix synthesis in ankle cartilage, while Eger and colleagues found that ankle explants were less catabolically responsive to interleukin-1beta and rebounded more strongly after cytokine removal (15–17). These findings define a distinct homeostatic and catabolic baseline for the ankle joint.

The ankle matrix also creates a different exposure environment for soluble mediators. Fetter and colleagues showed that ankle and knee cartilage differ in composition and transport properties, and Quinn and colleagues demonstrated depth-associated adaptations in adult knee and ankle cell-matrix organization (18, 19). Additional studies of cartilage degeneration and chondron morphology reinforce the idea that the ankle surface is organized around a different mechanical and biological role (20, 21).

Mechanical injury response extends this joint-specific logic. Joint-dependent impact studies and recent shear-strain experiments suggest that ankle and knee cartilage do not respond identically to traumatic mechanical loading (22, 23). The immediate implication for this review is not a new treatment claim. It is a stronger organizing principle: ankle OA requires a dedicated phenotyping vocabulary because its tissue biology, matrix environment, and load history are not interchangeable with those of the knee.

## Synovial and molecular phenotyping of ankle osteoarthritis

The molecular evidence for ankle OA is best read against the broader role of synovitis in OA pathogenesis. Synovial inflammation can shape pain, structural progression, and joint-tissue crosstalk in OA, which makes the ankle synovium a biologically plausible disease-informative tissue rather than a passive bystander (24). In acute intra-articular ankle fracture, Furman and colleagues reported increased synovitis, a trend toward greater CD68+ macrophage infiltration, and elevated synovial-fluid cytokines and chemokines (25).

Early or less advanced ankle disease shows a broader remodeling profile. Schmal and colleagues found that aggrecan and bone morphogenetic protein signals in ankle lavage were related to symptoms, function, radiographic stage, or cartilage damage, and a related analysis showed that aggrecan, bFGF, BMP-7, and CD105 varied with age and OA stage. Candidate local-fluid studies then reported severity-linked alpha-MSH and ghrelin patterns in post-traumatic ankle OA (26, 35–37). These studies support local sampling, but they remain cross-sectional candidate-marker evidence.

WNT-related biology gives the molecular domain a stronger pathway anchor. WNT signaling has long been discussed as a therapeutic and mechanistic target in OA, and recent ankle synovial transcriptomic studies report distinct gene-expression profiles enriched for extracellular-matrix remodeling, tissue morphogenesis, immune activity, and WNT-related dysregulation (38–40). The post-traumatic ankle OA signal is especially important because it suggests that fracture and chronic PTOA may share a molecular fingerprint that differs from non-traumatic ankle OA.

The broader OA field is moving from single biomarkers toward endotypes and phenotypes. Biochemical-marker clustering, phenotype reviews, and recent precision-medicine discussions all argue that OA subgroups should be defined by convergent clinical, structural, and molecular data rather than by radiographs alone (41–43). This logic fits ankle OA particularly well because its molecular signals are interpretable only when injury timing, alignment, and function are measured at the same time.

Taken together, the molecular studies in Table 1 move ankle OA phenotyping beyond a single-biomarker search. They identify a sequence of measurable states: acute post-injury inflammation, early remodeling signals, severity-linked local-fluid markers, and chronic synovial transcriptomic programs. This sequence creates a molecular layer that can be aligned with WBCT and digital functional measurements.

## Weight-bearing structural phenotyping from WBCT to distance maps

If molecular studies show that ankle OA is more than cartilage wear, WBCT studies show that it is more than a flat-plane radiographic deformity. A systematic review of clinical weightbearing CT studies supports its value for foot and ankle assessment, and Kim and colleagues demonstrated that varus ankle OA commonly includes abnormal internal rotation of the talus in the axial plane (27, 28). This rotational component helps explain why conventional radiographs can under-describe the load-bearing phenotype.

The ankle also functions within a peritalar and whole-limb alignment chain. Krahenbuhl and colleagues found valgus subtalar compensation in varus ankle OA, Kvarda and colleagues showed that three-dimensional WBCT measurements were more reliable than two-dimensional measures in end-stage post-traumatic ankle OA, and additional WBCT studies linked hindfoot alignment with knee or lower-limb coronal alignment (29, 30, 44, 45). These observations make hindfoot and midfoot posture part of the ankle OA phenotype.

Peritalar structure is also relevant after surgery. WBCT studies of total ankle replacement and deformity correction suggest that subtalar alignment and three-dimensional deformity may change or persist after arthroplasty, depending on the correction strategy and residual load path (46, 47). This matters because structural phenotyping should describe not only the preoperative deformity but also the postoperative mechanical environment.

Automation is important because multidimensional structural assessment cannot scale if it depends entirely on manual measurement. Early software-based WBCT angular measurement saved time but did not fully match manual measurement, whereas later validation work reported stronger agreement with manual measurement while preserving efficiency (31, 48). Computational joint-space and distance-map methods further extend WBCT from angles to digital joint-contact signatures (49–51).

Classification remains a practical bridge between imaging and treatment research. Early-stage alignment studies and reliability analyses of post-traumatic ankle OA classification show that radiographic staging and alignment categories require careful standardization (52, 53). For this review, the structural phenotype has three practical components: axial tibiotalar rotation, peritalar compensation, and spatial joint-contact distribution. These components are summarized with the imaging evidence in Table 1.

## Digital functional phenotyping from gait and plantar pressure to wearable trajectories

Digital functional phenotyping asks how ankle OA behaves during walking, loading, and recovery. Valderrabano and colleagues established that unilateral post-traumatic ankle OA impairs spatiotemporal variables, three-plane motion, joint moments, and power, with partial recovery after total ankle arthroplasty over 12 months (32). Dynamic plantar pressure studies then showed that painful post-traumatic end-stage ankle OA redistributes load across the foot and that longer walking trials can capture treatment-related pressure features (33, 34).

COP trajectories condense rollover behavior into a compact digital metric. Zeininger and colleagues found that symptomatic ankle OA shortened the proximodistal COP path and altered COP position; after total ankle arthroplasty, bilateral COP progression increased and persisted for more than 2 years (54). COP therefore functions as a recovery trajectory marker as well as a baseline load signature.

Treatment comparisons provide the functional validation context for phenotyping. Supramalleolar osteotomy indications and systematic reviews support joint-preserving correction in selected varus ankle OA, while comparative reviews of total ankle arthroplasty and arthrodesis show that salvage choices differ in clinical outcomes and gait-biomechanical effects (55–58). These treatment studies do not yet define precision subtypes, but they define the outcomes that future phenotypes must predict.

Ankle-specific ambulatory evidence now permits a more direct extension beyond hip- and knee-focused wearable literature. In a prospective surgical cohort, step-activity monitoring before arthrodesis or arthroplasty and at 6, 12, 24, and 36 months showed longitudinal improvement, particularly in higher-activity stepping, and objective activity changes were associated with patient-reported outcomes (59). A separate ankle OA case-control study used an accelerometer-based monitor and found substantially lower daily step counts and lower attainment of activity targets than in controls (60). These studies establish feasibility and clinical discrimination, but they do not establish universal wearable thresholds or treatment-specific recovery classes. Future protocols should therefore align device wear windows with laboratory gait, plantar pressure, COP, PROM, and WBCT visits; preserve device, firmware, non-wear, and walking-bout metadata; and evaluate whether activity volume, intensity, regularity, and recovery slopes add information beyond conventional follow-up.

The functional domain completes the proposed molecular-biomechanical model shown in Figure 1. Synovial markers describe the local disease environment, WBCT and distance maps describe the weight-bearing structure, and gait, plantar pressure, COP, and wearable trajectories describe how the patient uses the limb. These signals should not be assumed to cohere in every patient. Repeated same-patient measurement is required to determine whether multimodal patterns are reproducible, prognostic, and responsive to treatment rather than parallel observations collected in different cohorts.

## Discussion: phenotype-to-decision framework and validation pathway

### Operational terminology and provisional phenotype axes

Within this review, phenotype denotes an observable pattern defined by prespecified molecular, weight-bearing structural, digital functional, and clinical measurements. Endotype is reserved for a reproducible subgroup supported by a distinct biological mechanism and, ideally, differential prognosis or treatment response. Subtype is used only as a mechanism-neutral umbrella term for an empirically separable group, including etiologic or morphological groupings. These operational definitions prevent a molecular association, imaging pattern, or clustering result from being interpreted prematurely as a mechanistic endotype.

Accordingly, inflammatory-dominant, malalignment-dominant, post-traumatic or instability-dominant, and end-stage structural collapse are four provisional, non-mutually-exclusive baseline research phenotypes. One patient may express more than one axis, and the dominant signal may change with disease stage or intervention. Post-surgical recovery is not a fifth baseline class; it is a longitudinal response trajectory conditioned by the preoperative phenotype, treatment, rehabilitation, and complications. The labels should be promoted to clinical classes or mechanistic endotypes only after multidomain cohorts show reproducibility, biological coherence, prognostic value, and treatment interaction (41–43). The four axes are not equally eligible for that promotion. Inflammatory-dominant and post-traumatic or instability-dominant disease each have a candidate mechanism attached to them, in the form of synovial inflammatory and WNT-related transcriptomic programmes, and are therefore endotype candidates that multidomain cohorts could in principle refine into mechanistic subgroups. Malalignment-dominant disease and end-stage structural collapse describe load paths and structural states rather than biological mechanisms; they are expected to remain phenotypes, and their value lies in conditioning treatment response and in modifying how the molecular axes should be interpreted, not in becoming endotypes themselves.

The inflammatory-dominant phenotype captures patients in whom acute injury biology, synovitis, local cytokines, candidate fluid markers, or WNT-related synovial programs are central signals. Its value is to define sampling windows and trial-enrichment strategies for early post-injury and early post-traumatic OA research (24–26, 35).

The malalignment-dominant phenotype captures the structural load path. It is defined by varus or valgus alignment, talar tilt, axial talar rotation, subtalar or midtarsal compensation, and distance-map asymmetry. This phenotype makes alignment-sensitive planning more explicit by separating tibiotalar deformity from compensatory or decompensated peritalar posture (27–30).

The post-traumatic or instability-dominant phenotype requires a stricter definition than injury history alone. Because most ankle OA is post-traumatic, a history of fracture or sprain carries little discriminative value and cannot by itself define a subgroup (1, 3). We therefore define this phenotype by an *active* post-traumatic signature rather than by a past event: a persisting PTOA-associated synovial molecular programme, exemplified by the WNT7B- and IL11-enriched fingerprint shared by fracture and post-traumatic synovium, and/or demonstrable mechanical instability with rotational malalignment, residual articular step-off, or asymmetric load progression (4, 39). Its purpose is to separate patients whose disease is still driven by an active post-traumatic biological and mechanical process from those in whom the traumatic insult is historical and the disease has become a burnt-out, predominantly mechanical one. Radiographic staging cannot make this distinction, and the distinction plausibly conditions the response to biological, corrective, and salvage treatments.

The end-stage structural collapse phenotype integrates severe joint-space loss, distance-map collapse, hindfoot compensation, reduced gait power, plantar offloading, and shortened COP progression. Its purpose is to improve salvage-decision research by asking how residual alignment, peritalar compensation, and functional load capacity modify expectations after arthroplasty or fusion (32–34, 54).

### Longitudinal post-surgical recovery trajectories

Post-surgical recovery should be operationalized as within-patient change rather than as a generic postoperative time label. A common schedule should include a preoperative assessment and follow-up at 3, 6, 12, and 24 months, with longer surveillance when revision or salvage is relevant. Each visit should collect the same ankle-specific PROM and record complications, rehabilitation exposure, and subsequent procedures. Functional assessment should combine COP progression, regional plantar pressure, gait when feasible, and wearable-derived step counts, cadence bands, walking-bout duration, and day-to-day regularity. WBCT alignment and distance maps can anchor the preoperative state and document clinically indicated residual or recurrent structural abnormalities (32–34, 46, 47, 54). Serial ankle-specific step-monitor data demonstrate that objective activity may continue to change after PROMs have plateaued, supporting repeated trajectory measurement rather than a single postoperative snapshot (59).

Candidate trajectory classes include early sustained recovery, delayed recovery, persistent impairment, and secondary deterioration after initial improvement. Early sustained recovery would show improvement by 3–6 months, attainment of a prespecified PROM minimal important difference, and maintained objective gains through 24 months. Delayed recovery would reach comparable benchmarks only at 12–24 months; persistent impairment would remain below both PROM and functional benchmarks; and secondary deterioration would show an initial gain followed by recurrent asymmetry, declining activity, worsening PROMs, or a new adverse event. For initial research, COP progression reaching at least 80% of the contralateral value by 12 months and a limb asymmetry index below 10% by 24 months can be tested as candidate benchmarks. They are not validated clinical cutoffs. Their interpretation must account for contralateral disease and be calibrated against healthy reference ranges, procedure-specific expectations, PROM change, complications, and patient-valued recovery.

### Evidence maturity and limitations

A pragmatic narrative maturity hierarchy, rather than a formal GRADE or risk-of-bias score, helps distinguish what the present literature can and cannot support. Comparative ankle-knee cartilage biology is the well-established tier because multiple studies converge on joint-dependent matrix organization, metabolism, transport, and injury responses, although some evidence remains *ex vivo* or non-human (13–23). Human synovial-fluid associations, synovial transcriptomics, automated WBCT, three-dimensional alignment, distance maps, and laboratory gait and plantar pressure analysis are promising but preliminary: they demonstrate biological or measurement feasibility, not stable endotypes, prognostic thresholds, or differential treatment effects (25–39, 44–51). COP recovery trajectories and ankle-specific wearable monitoring remain exploratory, because they rest on single-center cohorts that have not been paired with molecular or structural data, and the wider wearable and AI literature on which they build remains hip- and knee-derived (54, 59–62). These tiers correspond one-to-one with the maturity labels reported for every study in Table 1.

The principal risks of bias arise from small or selected samples, single-center recruitment, cross-sectional or retrospective designs, incomplete blinding or reliability reporting, and analyses confined to one modality. Synovial studies often depend on surgically obtained tissue, creating selection and disease-stage bias. WBCT and distance-map reports commonly emphasize reliability or feasibility rather than longitudinal clinical validity. Functional studies may be confounded by contralateral disease, treatment indication, rehabilitation exposure, device adherence, and variable follow-up. Heterogeneous etiologies, disease stages, procedures, outcome definitions, and visit schedules further limit comparison. Table 1 now reports the design and sample of every study together with an explicit maturity tier and its principal limitation, so that these constraints remain visible at the point of citation; the heterogeneity of the included designs nonetheless does not support a defensible pooled quality score. No identified study repeatedly measured all three domains in the same patients within a longitudinal response model. Independent threshold testing and external model evaluation are also uncommon; causal, prognostic, and treatment-selection claims should therefore remain explicitly provisional.

### Prospective validation and multimodal modeling

#### The minimum same-patient dataset

Multimodal phenotyping is testable only if the same individuals contribute all three domains at defined time points, so we specify a minimum dataset rather than an open-ended wish list. The molecular domain comprises synovial fluid or synovial tissue whenever a procedure makes it obtainable, together with a mandatory serum panel so that a less invasive proxy exists for every participant. The structural domain comprises WBCT with automated three-dimensional alignment and a standardized distance-map output. The functional domain comprises COP progression and regional plantar pressure in all participants, laboratory gait where a motion laboratory is available, and continuous wearable step, cadence, and walking-bout data in all participants. The clinical domain comprises etiology, injury timing, radiographic stage, deformity, pain, AOS, and routine covariates. Assessments are anchored at baseline and at 3, 6, 12, and 24 months. Only the molecular domain is conditional on surgical access; every other element is mandatory. Fixing the dataset in this way defines the analysable unit of the cohort and makes the incremental value of each domain estimable rather than opportunistic.

#### A structural limitation follows directly from this design

Synovial tissue and synovial fluid can be obtained in most patients only when surgery is performed. The molecular domain is therefore systematically missing in precisely the non-operative, early, and joint-preserving patients for whom stratification would be most valuable, and this missingness is not random: it is conditional on disease severity and treatment indication. Serum biomarkers are included in the minimum dataset as a mandatory, less invasive proxy; any molecular-domain model must be evaluated under missing-not-at-random assumptions with explicit sensitivity analysis rather than by complete-case analysis restricted to surgically treated patients (63).

Validation should proceed through a prospective multicenter cohort with a protocol-specified primary outcome: change in the Ankle Osteoarthritis Scale (AOS) total score from baseline to 24 months. AOS should be collected at baseline and at 3, 6, 12, and 24 months in a validated language version. Secondary outcomes should include pain and function trajectories, clinically important PROM response, complications, time to revision, conversion, or other salvage surgery, WBCT and distance-map change, gait, COP and plantar pressure recovery, and wearable-derived activity trajectories. A published estimate of 5.81 points for the AOS minimal important difference (MID, often termed MCID) in surgically treated end-stage ankle arthritis provides an initial planning value, but it varied with age and baseline status (64). The responder rule should therefore be prespecified, anchored to a credible patient-reported external criterion, and triangulated with distribution-based estimates; a published MID should be transferred only when instrument, language, severity, treatment, and follow-up are comparable (65). That estimate was derived in surgically treated end-stage ankle arthritis and, by the transfer rule just stated, should not be applied unchanged to the joint-preserving or non-operative strata of the cohort. A supramalleolar-osteotomy or rehabilitation stratum requires its own anchor-based MID estimated within the cohort, and the responder definition must be prespecified separately for each treatment stratum (65).

Sample size should be determined by the primary estimand and the most demanding prespecified prediction analysis, not by an arbitrary fixed target or a simple events-per-variable rule. The longitudinal AOS calculation should incorporate outcome variance, within-patient correlation, center clustering, planned interactions, and 15–20% attrition. Prediction-model calculations should also specify candidate predictor degrees of freedom, anticipated model fit, event incidence or censoring for time-to-event outcomes, and target shrinkage and calibration precision (66). A feasibility phase or existing registry should supply these inputs; the resulting multicenter cohort will likely require several hundred participants rather than the tens typical of current single-center studies. Linear mixed-effects models with participant and center effects should analyze repeated AOS and functional measures. Cox models with center frailty can address revision or salvage after assumptions are checked, with flexible parametric or competing-risk methods used when needed. Procedure pathways should be stratified or represented explicitly rather than pooled as exchangeable treatments (55–58).

Phenotype discovery and outcome prediction should be separated analytically. For discovery, consensus or sparse multiview clustering can combine assay-normalized molecular features, standardized WBCT and distance-map descriptors, and gait, COP, plantar pressure, and wearable summaries; cluster stability must be assessed by resampling and replication across centers (41–43). Latent-class mixed models can test recovery trajectories because they use repeated measurements rather than reducing recovery to one visit. For prediction, penalized regression should provide the transparent benchmark. Random forests or gradient-boosted trees can be secondary nonlinear comparators when participant and event counts support them; a multimodal neural network should not be the default for an early ankle OA cohort. A practical integration strategy is multiblock or late fusion: quality-control and, when necessary, reduce each domain within training data, then combine domain-level features with a prespecified clinical base model containing etiology, radiographic stage, pain, deformity, and routine covariates. This design tests the incremental value of each domain instead of attributing performance to an opaque concatenation of variables.

Such modeling depends more on coordination than algorithmic novelty. Before recruitment, investigators should lock a common data dictionary, index dates and visit windows, laboratory procedures, WBCT reconstruction and segmentation rules, distance-map definitions, wearable device and firmware metadata, and central quality-control criteria. Center, scanner, assay-batch, and device effects should be retained as metadata, and validation-center data must not influence training-stage normalization. Reasons for missingness should distinguish non-collection, contraindication, tissue unavailable because surgery was not performed, assay or device failure, and wearable non-wear. Multiple imputation should respect the multilevel longitudinal structure and occur within each resampling split, with sensitivity analyses for departures from missing-at-random assumptions (63). Performance should be reported as optimism-corrected prediction error and R-squared for continuous outcomes or time-dependent Brier score and discrimination for survival outcomes, together with calibration plots, intercept, and slope. Nested resampling should precede leave-one-center-out internal-external evaluation and testing of a locked model in a later geographic or temporal cohort. Coefficients, domain scores, calibrated individual predictions, and clinically recognizable cluster profiles should lead interpretation; feature-attribution plots can support error analysis but cannot establish mechanism. Reporting should follow TRIPOD+AI (67).

## Conclusion

Ankle OA is best studied as a joint-specific molecular-biomechanical disease state rather than radiographic wear alone. Current evidence supports an ankle-specific biological foundation, measurable synovial signals, three-dimensional weight-bearing structural signatures, and digital functional load patterns. These data justify four provisional, non-mutually-exclusive baseline research phenotypes and a separate longitudinal post-surgical recovery trajectory, but not validated clinical classes or mechanistic endotypes. The next step is a prospective multicenter cohort using repeated same-patient measurement, a 24-month ankle-specific PROM primary outcome, prespecified MID and candidate recovery thresholds, transparent multimodal integration, calibration, and external evaluation. Until those tests are completed, the framework should organize cohort design and treatment-response questions rather than direct individual treatment.

## References

[1] Saltzman CL Salamon ML Blanchard GM Huff T Hayes A Buckwalter JA . Epidemiology of ankle arthritis: report of a consecutive series of 639 patients from a tertiary orthopaedic center. Iowa Orthop J. (2005) 25:44–6. 16089071 PMC1888779

[2] Glazebrook M Daniels T Younger A Foote CJ Penner M Wing K . Comparison of health-related quality of life between patients with end-stage ankle and hip arthrosis. J Bone Joint Surg Am. (2008) 90:499–505. doi: 10.2106/JBJS.F.01299 18310699

[3] Valderrabano V Horisberger M Russell I Dougall H Hintermann B. Etiology of ankle osteoarthritis. Clin Orthop Relat Res. (2009) 467:1800–6. doi: 10.1007/s11999-008-0543-6 18830791 PMC2690733

[4] Horisberger M Valderrabano V Hintermann B. Posttraumatic ankle osteoarthritis after ankle-related fractures. J Orthop Trauma. (2009) 23:60–7. doi: 10.1097/BOT.0b013e31818915d9 19104305

[5] Anderson DD Ledoux WR Lenz AL Wilken J Easley ME de Cesar Netto C. Ankle osteoarthritis: Toward new understanding and opportunities for prevention and intervention. J Orthop Res. (2024) 42:2613–22. doi: 10.1002/jor.25973 39269016 PMC11981820

[6] Khlopas H Khlopas A Samuel LT Ohliger E Sultan AA Chughtai M . Current concepts in osteoarthritis of the ankle: review. Surg Technol Int. (2019) 35:280–94. 31237341

[7] Thomas AC Hubbard-Turner T Wikstrom EA Palmieri-Smith RM. Epidemiology of posttraumatic osteoarthritis. J Athl Train. (2017) 52:491–6. doi: 10.4085/1062-6050-51.5.08 27145096 PMC5488839

[8] Gribble PA Bleakley CM Caulfield BM Docherty CL Fourchet F Fong DT . 2016 consensus statement of the International Ankle Consortium: prevalence, impact and long-term consequences of lateral ankle sprains. Br J Sports Med. (2016) 50:1493–5. doi: 10.1136/bjsports-2016-096188 27259750

[9] Verhage SM Krijnen P Schipper IB Hoogendoorn JM. Persistent postoperative step-off of the posterior malleolus leads to higher incidence of post-traumatic osteoarthritis in trimalleolar fractures. Arch Orthop Trauma Surg. (2019) 139:323–9. doi: 10.1007/s00402-018-3056-0 30430238 PMC6394475

[10] Swierstra BA van Enst WA. The prognosis of ankle fractures: a systematic review. EFORT Open Rev. (2022) 7:692–700. doi: 10.1530/EOR-22-0065 36287098 PMC9619393

[11] Okoye AU Houchen-Wolloff L Mangwani J Akram N Laparidou D Nelson D . A systematic review: Radiological findings at a minimum of 3 years follow-up for unstable ankle fractures in adults treated with surgery. Foot. (2024) 61:102143. doi: 10.1016/j.foot.2024.102143 39612557

[12] Fonkoue L Sarr L Muluem KO Gueye AB Dembele B Fon C . Early posttraumatic ankle osteoarthritis following ankle fracture-dislocations in a sub-Saharan African setting. Orthop Traumatol Surg Res. (2021) 107:102996. doi: 10.1016/j.otsr.2021.102996 34198007

[13] Huch K Kuettner KE Dieppe P. Osteoarthritis in ankle and knee joints. Semin Arthritis Rheum. (1997) 26:667–74. doi: 10.1016/S0049-0172(97)80002-9 9062947

[14] Cole AA Kuettner KE. Molecular basis for differences between human joints. Cell Mol Life Sci. (2002) 59:19–26. doi: 10.1007/s00018-002-8401-2 11846028 PMC11337503

[15] Kang Y Koepp H Cole AA Kuettner KE Homandberg GA. Cultured human ankle and knee cartilage differ in susceptibility to damage mediated by fibronectin fragments. J Orthop Res. (1998) 16:551–6. doi: 10.1002/jor.1100160505 9820277

[16] Huch K. Knee and ankle: human joints with different susceptibility to osteoarthritis reveal different cartilage cellularity and matrix synthesis *in vitro*. Arch Orthop Trauma Surg. (2001) 121:301–6. doi: 10.1007/s004020000225 11482459

[17] Eger W Schumacher BL Mollenhauer J Kuettner KE Cole AA. Human knee and ankle cartilage explants: catabolic differences. J Orthop Res. (2002) 20:526–34. doi: 10.1016/S0736-0266(01)00125-5 12038627

[18] Fetter NL Leddy HA Guilak F Nunley JA. Composition and transport properties of human ankle and knee cartilage. J Orthop Res. (2006) 24:211–9. doi: 10.1002/jor.20029 16435350

[19] Quinn TM Häuselmann HJ Shintani N Hunziker EB. Cell and matrix morphology in articular cartilage from adult human knee and ankle joints suggests depth-associated adaptations to biomechanical and anatomical roles. Osteoarthritis Cartilage. (2013) 21:1904–12. doi: 10.1016/j.joca.2013.09.011 24455780

[20] Kuettner KE Cole AA. Cartilage degeneration in different human joints. Osteoarthritis Cartilage. (2005) 13:93–103. doi: 10.1016/j.joca.2004.11.006 15694570

[21] Schumacher BL Su JL Lindley KM Kuettner KE Cole AA. Horizontally oriented clusters of multiple chondrons in the superficial zone of ankle, but not knee articular cartilage. Anat Rec. (2002) 266:241–8. doi: 10.1002/ar.10063 11920387

[22] Novakofski KD Berg LC Bronzini I Bonnevie ED Poland SG Bonassar LJ . Joint-dependent response to impact and implications for post-traumatic osteoarthritis. Osteoarthritis Cartilage. (2015) 23:1130–7. doi: 10.1016/j.joca.2015.02.023 25725390 PMC4778978

[23] Ayala S Delco ML Fortier LA Cohen I Bonassar LJ. Articular chondrocytes from the knee and ankle have different sensitivities to shear strain. J Biomech. (2026) 200:113237. doi: 10.1016/j.jbiomech.2026.113237 41797056

[24] Scanzello CR Goldring SR. The role of synovitis in osteoarthritis pathogenesis. Bone. (2012) 51:249–57. doi: 10.1016/j.bone.2012.02.012 22387238 PMC3372675

[25] Furman BD Kimmerling KA Zura RD Reilly RM Zlowodzki MP Huebner JL . Articular ankle fracture results in increased synovitis, synovial macrophage infiltration, and synovial fluid concentrations of inflammatory cytokines and chemokines. Arthritis Rheumatol. (2015) 67:1234–9. doi: 10.1002/art.39064 25707992 PMC4819001

[26] Schmal H Salzmann GM Langenmair ER Henkelmann R Südkamp NP Niemeyer P. Biochemical characterization of early osteoarthritis in the ankle. ScientificWorldJournal. (2014) 2014:434802. doi: 10.1155/2014/434802 24696644 PMC3947760

[27] Li J Fang M Van Oevelen A Peiffer M Audenaert E Burssens A . Diagnostic applications and benefits of weightbearing CT in the foot and ankle: A systematic review of clinical studies. Foot Ankle Surg. (2024) 30:7–20. doi: 10.1016/j.fas.2023.09.001 37704542

[28] Kim JB Yi Y Kim JY Cho JH Kwon MS Choi SH . Weight-bearing computed tomography findings in varus ankle osteoarthritis: abnormal internal rotation of the talus in the axial plane. Skeletal Radiol. (2017) 46:1071–80. doi: 10.1007/s00256-017-2655-0 28432396

[29] Krähenbühl N Siegler L Deforth M Zwicky L Hintermann B Knupp M. Subtalar joint alignment in ankle osteoarthritis. Foot Ankle Surg. (2019) 25:143–9. doi: 10.1016/j.fas.2017.10.004 29409290

[30] Kvarda P Heisler L Krähenbühl N Steiner CS Ruiz R Susdorf R . 3D Assessment in posttraumatic ankle osteoarthritis. Foot Ankle Int. (2021) 42:200–14. doi: 10.1177/1071100720961315 33073607

[31] Richter M Schilke R Duerr F Zech S Andreas Meissner S Naef I. Automatic software-based 3D-angular measurement for Weight-Bearing CT (WBCT) provides different angles than measurement by hand. Foot Ankle Surg. (2022) 28:863–71. doi: 10.1016/j.fas.2021.11.010 34876354

[32] Valderrabano V Nigg BM von Tscharner V Stefanyshyn DJ Goepfert B Hintermann B. Gait analysis in ankle osteoarthritis and total ankle replacement. Clin Biomech. (2007) 22:894–904. doi: 10.1016/j.clinbiomech.2007.05.003 17604886

[33] Horisberger M Hintermann B Valderrabano V. Alterations of plantar pressure distribution in posttraumatic end-stage ankle osteoarthritis. Clin Biomech. (2009) 24:303–7. doi: 10.1016/j.clinbiomech.2008.12.005 19150745

[34] Rouhani H Crevoisier X Favre J Aminian K. Outcome evaluation of ankle osteoarthritis treatments: plantar pressure analysis during relatively long-distance walking. Clin Biomech. (2011) 26:397–404. doi: 10.1016/j.clinbiomech.2010.11.011 21168247

[35] Schmal H Henkelmann R Mehlhorn AT Reising K Bode G Südkamp NP . Synovial cytokine expression in ankle osteoarthritis depends on age and stage. Knee Surg Sports Traumatol Arthrosc. (2015) 23:1359–67. doi: 10.1007/s00167-013-2719-1 24141892

[36] Liu G Chen Y Wang G Niu J. Decreased Synovial Fluid α-Melanocyte-Stimulating-Hormone (α-MSH) Levels Reflect Disease Severity in Patients with Posttraumatic Ankle Osteoarthritis. Clin Lab. (2016) 62:1491–500. doi: 10.7754/Clin.Lab.2016.151222 28164623

[37] Zou YC Li HH Yang GG Yin HD Cai DZ Liu G. Attenuated levels of ghrelin in synovial fluid is related to the disease severity of ankle post-traumatic osteoarthritis. Biofactors. (2019) 45:463–70. doi: 10.1002/biof.1490 30697843

[38] Matthias J Buckley S David M Ackert-Bicknell CL Metzl JA Moon DK . Synovial Dysregulation in Ankle Osteoarthritis: Molecular Insights and Pathogenetic Pathways. J Orthop Res. (2025) 43:1736–47. doi: 10.1002/jor.70016 40605342 PMC13495539

[39] Matthias J David MA Buckley SE Stark SD Austin NA Zona NE . The Synovial Fingerprint of Ankle Fractures: A Central Role for WNT Pathway Activation in Posttraumatic Ankle Osteoarthritis. Foot Ankle Int. (2026) 47:662–73. doi: 10.1177/10711007251413557 41871107

[40] Blom AB van Lent PL van der Kraan PM van den Berg WB. To seek shelter from the WNT in osteoarthritis? WNT-signaling as a target for osteoarthritis therapy. Curr Drug Targets. (2010) 11:620–9. doi: 10.2174/138945010791011901 20199388

[41] Angelini F Widera P Mobasheri A Blair J Struglics A Uebelhoer M . Osteoarthritis endotype discovery via clustering of biochemical marker data. Ann Rheum Dis. (2022) 81:666–75. doi: 10.1136/annrheumdis-2021-221763 35246457

[42] Mobasheri A Saarakkala S Finnilä M Karsdal MA Bay-Jensen AC van Spil WE. Recent advances in understanding the phenotypes of osteoarthritis. F1000Res. (2019) 8:2091. doi: 10.12688/f1000research.20575.1 31885861 PMC6913225

[43] Vaishya R Wamuyu EN Vaish A Handa R Kumar D. Osteoarthritis phenotypes: advancing precision medicine through clinical, structural, and molecular stratification. Int Orthop. (2026) 50:1233–47. doi: 10.1007/s00264-026-06845-9 42141125

[44] Dagneaux L Dufrenot M Bernasconi A Bedard NA de Cesar Netto C Lintz F. Three-Dimensional Biometrics to Correlate Hindfoot and Knee Coronal Alignments Using Modern Weightbearing Imaging. Foot Ankle Int. (2020) 41:1411–8. doi: 10.1177/1071100720938333 32698631

[45] Burssens ABM Buedts K Barg A Vluggen E Demey P Saltzman CL . Is Lower-limb Alignment Associated with Hindfoot Deformity in the Coronal Plane? A Weightbearing CT Analysis. Clin Orthop Relat Res. (2020) 478:154–68. doi: 10.1097/CORR.0000000000001067 31809289 PMC7000051

[46] Kvarda P Siegler L Burssens A Susdorf R Ruiz R Hintermann B. Effect of total ankle replacement on the 3-dimensional subtalar joint alignment in varus ankle osteoarthritis. Foot Ankle Surg. (2023) 29:424–9. doi: 10.1016/j.fas.2023.05.009 37296030

[47] VandeLune C Barbachan Mansur NS Iehl C Tazegul T Ahrenholz SJ de Carvalho KAM . Deformity Correction in Ankle Osteoarthritis Using a Lateral Trans-Fibular Total Ankle Replacement: A Weight-Bearing CT Assessment. Iowa Orthop J. (2022) 42:36–46. 36601228 PMC9769357

[48] Richter M Zech S Naef I Duerr F Schilke R. Automatic software-based 3D-angular measurement for weight-bearing CT (WBCT) is valid. Foot Ankle Surg. (2024) 30:417–22. doi: 10.1016/j.fas.2024.02.016 38448344

[49] Tazegul TE Anderson DD Barbachan Mansur NS Kajimura Chinelati RM Iehl C VandeLune C . An Objective Computational Method to Quantify Ankle Osteoarthritis From Low-Dose Weightbearing Computed Tomography. Foot Ankle Orthop. (2022) 7:24730114221116805. doi: 10.1177/24730114221116805 36478960 PMC9720551

[50] Eatough ZJ Peterson AC Lisonbee RJ Miyamoto T Tanaka Y Saltzman CL . Static posture weightbearing joint angle differences in patients with varus ankle osteoarthritis. Gait Posture. (2024) 112:33–9. doi: 10.1016/j.gaitpost.2024.05.003 38729081 PMC11234790

[51] Efrima B Barbero A Benady A Halimi YG Dahmen J Kerkhoffs GMMJ . The Battleship technique: A reliable method to quantify intraarticular distance maps patterns and correlate hindfoot alignment. J Exp Orthop. (2025) 12:e70190. doi: 10.1002/jeo2.70190 40059955 PMC11888772

[52] Lee WC Moon JS Lee HS Lee K. Alignment of ankle and hindfoot in early stage ankle osteoarthritis. Foot Ankle Int. (2011) 32:693–9. doi: 10.3113/FAI.2011.0693 21972764

[53] Claessen FM Meijer DT van den Bekerom MP Gevers Deynoot BD Mallee WH Doornberg JN . Reliability of classification for post-traumatic ankle osteoarthritis. Knee Surg Sports Traumatol Arthrosc. (2016) 24:1332–7. doi: 10.1007/s00167-015-3871-6 26611896 PMC4823329

[54] Zeininger A Schmitt D Hughes-Oliver C Queen RM. The effect of ankle osteoarthritis and total ankle arthroplasty on center of pressure position. J Orthop Res. (2021) 39:1245–52. doi: 10.1002/jor.24857 32918492

[55] Lee WC Moon JS Lee K Byun WJ Lee SH. Indications for supramalleolar osteotomy in patients with ankle osteoarthritis and varus deformity. J Bone Joint Surg Am. (2011) 93:1243–8. doi: 10.2106/JBJS.J.00249 21776578

[56] Christidis P Lampridis V Kalitsis C Kantas T Biniaris G Gougoulias N. Supramalleolar osteotomies for ankle arthritis: a systematic review. Arch Orthop Trauma Surg. (2023) 143:5549–64. doi: 10.1007/s00402-023-04867-1 37010603

[57] Shih CL Chen SJ Huang PJ. Clinical Outcomes of Total Ankle Arthroplasty Versus Ankle Arthrodesis for the Treatment of End-Stage Ankle Arthritis in the Last Decade: a Systematic Review and Meta-analysis. J Foot Ankle Surg. (2020) 59:1032–9. doi: 10.1053/j.jfas.2019.10.008 32709528

[58] Deleu PA Besse JL Naaim A Leemrijse T Birch I Devos Bevernage B . Change in gait biomechanics after total ankle replacement and ankle arthrodesis: a systematic review and meta-analysis. Clin Biomech (Bristol). (2020) 73:213–25. doi: 10.1016/j.clinbiomech.2020.01.015 32044672

[59] Shofer JB Ledoux WR Orendurff MS Hansen ST Davitt J Anderson JG . Step Activity After Surgical Treatment of Ankle Arthritis. J Bone Joint Surg Am. (2019) 101:1177–84. doi: 10.2106/JBJS.18.00511 31274719 PMC11883842

[60] Kimura S Yamaguchi S Ono Y Matsuura Y Sato Y Akagi R . Decreased physical activity in patients with ankle osteoarthritis. A case-control study comparing daily step counts. Foot Ankle Surg. (2022) 28:66–71. doi: 10.1016/j.fas.2021.01.011 33551322

[61] Khan ST Huffman N Li X Sharma A Winalski CS Ricchetti ET . Pain Assessment in Osteoarthritis: Present Practices and Future Prospects Including the Use of Biomarkers and Wearable Technologies, and AI-Driven Personalized Medicine. J Orthop Res. (2025) 43:1217–29. doi: 10.1002/jor.26082 40205648 PMC12159590

[62] Rose MJ Costello KE Eigenbrot S Torabian K Kumar D. Inertial Measurement Units and Application for Remote Health Care in Hip and Knee Osteoarthritis: Narrative Review. JMIR Rehabil Assist Technol. (2022) 9:e33521. doi: 10.2196/33521 35653180 PMC9204569

[63] Sterne JA White IR Carlin JB Spratt M Royston P Kenward MG . Multiple imputation for missing data in epidemiological and clinical research: potential and pitfalls. BMJ. (2009) 338:b2393. doi: 10.1136/bmj.b2393 19564179 PMC2714692

[64] Sutherland JM Albanese CM Wing K Zhang YJ Younger A Veljkovic A . Effect of Patient Demographics on Minimally Important Difference of Ankle Osteoarthritis Scale Among End-Stage Ankle Arthritis Patients. Foot Ankle Int. (2021) 42:624–32. doi: 10.1177/1071100720977842 33504200 PMC8127667

[65] Revicki D Hays RD Cella D Sloan J. Recommended methods for determining responsiveness and minimally important differences for patient-reported outcomes. J Clin Epidemiol. (2008) 61:102–9. doi: 10.1016/j.jclinepi.2007.03.012 18177782

[66] Riley RD Ensor J Snell KIE Harrell FE Jr Martin GP Reitsma JB . Calculating the sample size required for developing a clinical prediction model. BMJ. (2020) 368:m441. doi: 10.1136/bmj.m441 32188600

[67] Collins GS Moons KGM Dhiman P Riley RD Beam AL Van Calster B . TRIPOD+AI statement: updated guidance for reporting clinical prediction models that use regression or machine learning methods. BMJ. (2024) 385:e078378. doi: 10.1136/bmj-2023-078378 38626948 PMC11019967

